# Altered gene expression profiles in the lungs of benzo[*a*]pyrene-exposed mice in the presence of lipopolysaccharide-induced pulmonary inflammation^☆^

**DOI:** 10.1016/j.taap.2017.09.023

**Published:** 2017-12-01

**Authors:** Q. Shi, R.R. Fijten, D. Spina, Y. Riffo Vasquez, V.M. Arlt, R.W. Godschalk, F.J. Van Schooten

**Affiliations:** a Department of Toxicology & Pharmacology, NUTRIM School of Nutrition and Translational Research in Metabolism, Maastricht University, PO Box 616, 6200, MD, Maastricht, The Netherlands; b Sackler Institute of Pulmonary Pharmacology, Institute of Pharmaceutical Science, King's College London, 150 Stamford Street, London SE1 9NH, United Kingdom; c Analytical and Environmental Sciences Division, MRC-PHE Centre for Environmental & Health, King's College London, 150 Stamford Street, London SE1 9NH, United Kingdom

**Keywords:** RNA microarray, Lipopolysaccharide (LPS), Benzo[*a*]pyrene (B[*a*]P), Mouse lung

## Abstract

Patients with inflammatory lung diseases are often additionally exposed to polycyclic aromatic hydrocarbons like B[*a*]P and B[*a*]P-induced alterations in gene expression in these patients may contribute to the development of lung cancer. Mice were intra-nasally treated with lipopolysaccharide (LPS, 20 μg/mouse) to induce pulmonary inflammation and subsequently exposed to B[*a*]P (0.5 mg/mouse) by intratracheal instillation. Gene expression changes were analyzed in mouse lungs by RNA microarrays. Analysis of genes that are known to be involved in the cellular response to B[*a*]P indicated that LPS significantly inhibited gene expression of various enzymes linked to B[*a*]P metabolism, which was confirmed by phenotypic analyses of enzyme activity. Ultimately, these changes resulted in higher levels of B[*a*]P-DNA adducts in the lungs of mice exposed to B[*a*]P with prior LPS treatment compared to the lungs of mice exposed to B[*a*]P alone. Using principle component analysis (PCA), we found that of all the genes that were significantly altered in their expression, those that were able to separate the different exposure conditions were predominantly related to immune-response. Moreover, an overall analysis of differentially expressed genes indicated that cell-cell adhesion and cell-cell communication was inhibited in lungs of mice that received both B[*a*]P and LPS. Our results indicate that pulmonary inflammation increased the genotoxicity of B[*a*]P *via* inhibition of both phase I and II metabolism. Therefore, inflammation could be a critical contributor to B[*a*]P-induced carcinogenesis in humans.

## Highlights

• Inflammation inhibits the expression of genes involved in B[*a*]P metabolism.
• Changes in gene-expression do not necessarily reflect the changes in phenotype.
• Exposure to B[*a*]P affects the expression of genes involved in inflammation.
• Presence of inflammation enhances the formation of DNA adducts by B[*a*]P.

## 1 Introduction

During inflammation, a variety of inflammatory cells is recruited to the site of inflammation including macrophages, neutrophils and lymphocytes, which contribute to the establishment of an inflammatory micro-environment (King, 2015b). Within this micro-environment, a variety of inflammatory mediators or enzymes have been found, including reactive oxygen species (ROS), myeloperoxidase (MPO), β-glucuronidase, tumor necrosis factor (TNF-α) and interleukin (IL)-6, (Gabay, 2006, Gungor et al., 2007, Mittal et al., 2014, Shi et al., 2016a, Zelová and Hošek, 2013) that are suggested to additionally affect aryl hydrocarbon receptor (AhR) signaling. AhR signaling is important for the cellular response to many environmental carcinogens, such as polycyclic aromatic hydrocarbons (PAHs) like benzo[*a*]pyrene B[*a*]P. Indeed, individuals that are exposed to B[*a*]P in combination with pulmonary inflammation have an increased risk for developing lung cancer, for instance patients with chronic obstructive pulmonary disease (COPD) or emphysema (Gomes et al., 2014, King, 2015a).

Most patients with chronic lung inflammatory disease are also exposed to environmental genotoxicants, such as cigarette smoke, diesel exhaust or ambient air particulate matter (PM) of various sources that can carry chemical carcinogens (Grunig et al., 2014). For example, over 70% of cigarette smokers show inflammatory responses in the lung and especially those people have an increased risk of developing lung cancer (Walser et al., 2008). One major group of compounds in cigarette smoke are PAHs, with B[*a*]P as one of the best studied PAH that is classified as human carcinogen (Group1) by the International Agency for Research on Cancer (IARC) (Abdel-Shafy and Mansour, 2016, Ewa and Danuta, 2017). B[*a*]P crosses the cell membrane and forms a complex with AhR. After binding, the complex translocates into the nucleus where it interacts with the aryl hydrocarbon receptor nuclear translocator (ARNT) and stimulates B[*a*]P metabolism by inducing the gene expression of cytochrome P450 1A1 (*CYP1A1*) and 1B1 (*CYP1B1*) (Schults et al., 2014, Umannova et al., 2008). B[*a*]P exerts its mutagenic and carcinogenic properties only after metabolic activation, in which CYPs and epoxide hydrolase are involved; B[*a*]P is converted into the ultimate carcinogenic derivative B[*a*]P-7,8-dihydrodiol-9,10-epoxide (BPDE), which can covalently bind to DNA and form pre-mutagenic adducts, preferentially at guanine residues (*i.e.* 10-(deoxyguanosin-*N*^2^-yl)-7,8,9-trihydroxy-7,8,9,10-tetrahydro-B[*a*]P [dG-*N*^2^-BPDE]) (Arlt et al., 2008, Shi et al., 2016a, Shi et al., 2016b). However, the majority of reactive B[*a*]P intermediates is detoxified by phase II enzymes, which convert these intermediates into water soluble metabolites. Enzymes that are involved in this detoxification include glutathione-S-transferases (GSTs), UDP-glucuronosyltransferases (UGTs) and sulfotransferases (SULTs) (Ren et al., 2014, Zheng et al., 2002b). In case a reactive intermediate reaches the DNA and forms an adduct, B[*a*]P-induced DNA damage induces cell cycle arrest by activation of the tumor suppressor protein p53 to provide enough time for removal of the adducts by DNA repair enzymes. Regarding the bulky DNA lesions that are induced by B[*a*]P, nucleotide excision repair (NER) is the most important DNA repair pathway (Kucab et al., 2015, Verhofstad et al., 2010).

Several studies have shown that B[*a*]P is capable of inducing an inflammatory response but *vice versa* inflammatory mediators can also enhance B[*a*]P-induced genotoxicity (Smerdova et al., 2013, Uno and Makishima, 2009). For instance, after exposing rats intratracheally to B[*a*]P for 2 days, lung inflammation, edema, and epithelial damage was observed (Qamar et al., 2012), but additionally, exposure to B[*a*]P with concommittant activation of inflammatory-related pathways, largely increased B[*a*]P genotoxicity *via* various pathways. These pathways included signaling that was initiated by nuclear factor-κB (NF-κB), TNF-α, β-glucuronidase, hypoxia-inducible factor (HIF)-1, IL-6 and IL-8 (Ji et al., 2013, Patel and Gooderham, 2015, Schults et al., 2010, Shi et al., 2016a, Shi et al., 2017, Umannova et al., 2008). However, although studies have revealed that many inflammation-related mediators promote B[*a*]P genotoxicity, an overall view on how inflammation promotes B[*a*]P-induced DNA damage is still missing.

In previous studies (Arlt et al., 2015, Shi et al., 2016a) we used an animal model of inflammation (*i.e.* lipopolysaccharides (LPS)-treated mice) and showed that higher B[*a*]P-DNA adduct levels were observed in the lungs of LPS-treated mice that were additionally exposed to B[*a*]P relative to the lungs of mice exposed to B[*a*]P alone (Arlt et al., 2015, Shi et al., 2016a). In the current study, an RNA microarray analysis was performed (mouse whole genome arrays) on lung to get an overview of how inflammation can promote B[*a*]P-induced genotoxicity.

## 2 Materials and methods

### 2.1 Chemicals

Benzo[*a*]pyrene (B[*a*]P; CAS no. 50-32-8; purity > 96%) was purchased from Sigma-Aldrich (St Louis, MO). All other chemicals were of analytical purity or better.

### 2.2 Animal treatment

All animal experiments were approved by the Institutional Ethics Committee and conducted in accordance with the protocols approved by the Home Office under “The Animals (Scientific Procedures) Act (1986)” at King's College London (Arlt et al., 2015). C57B1/6 mice (male; approximately 8–10 weeks old, 20–25 g) were obtained from Charles River Laboratories and kept under controlled pathogen-free conditions and allowed food and water *ad libitum*. Mice were divided into four groups as follows: Group I: control group (*n* = 3), mice were nasally instilled with saline at day 0 and after 24 h, mice were intratracheally instilled with tricaprylin (25 μl/mouse); Group II: LPS group (*n* = 4), mice were nasally instilled with 20 μg LPS (*Escherichia coli*, serotype O55:B5; 1 mg/ml; dissolved in saline) at day 0 and after 24 h, mice were intratracheally instilled with tricaprylin (25 μl/mouse); Group III: B[*a*]P group (n = 4), mice were nasally instilled with saline at day 0 and after 24 h, mice were intratracheally instilled with B[*a*]P (0.5 mg in 25 μl tricaprylin/mouse); and Group IV: LPS&B[*a*]P group (n = 4), mice were nasally instilled with 20 μg LPS at day 0 and after 24 h, mice were intratracheally instilled with B[*a*]P (0.5 mg/mouse). All instillations were performed under anesthesia with isoflurane followed by injection of ketamine/zylazine (1 mg/0.166 mg per mouse). Mice were sacrificed at day 3 by intraperitoneal administration of anesthesia (2 g/kg body weight urethane). Lung tissue was collected and snap-frozen in liquid nitrogen. Samples were stored at − 80 °C until analysis. The selection of the 20 μg LPS/mouse is based on our previous study (Gungor et al., 2010) and the use of the 0.5 mg B[*a*]P/mouse is based on a study published by Dr. Hashimoto who investigated the *in vivo* mutagenicity of B[*a*]P in gpt delta mice (Hashimoto et al., 2005).

### 2.3 RNA isolation, purification and quality assessment

Total RNA was extracted from frozen lung tissue according to the manufacturer's instructions, using TRIzol reagent (Invitrogen, Breda, The Netherlands) and purified on columns using Qiagen RNeasy Micro Kit (Qiagen, Venlo, the Netherlands). RNA concentration and purity were assessed spectrometrically using a Nano Drop ND-1000 spectrophotometer (Isogen, IJsselstein, The Netherlands). RNA quality was assessed on an Agilent 2100 bioanalyzer (Agilent Technologies, Amsterdam, the Netherlands). Microarray hybridization experiments were only performed on RNA samples with a RNA Integrity Number (RIN) > 8.0.

### 2.4 Microarray processing

Total RNA (100 ng) was labelled by a Whole Transcript Sense Target Assay and hybridized to mouse whole-genome Affymetrix Gene 1.1 ST arrays targeting 21,115 unique genes (Affymetrix, Santa Clara, CA). Hybridization, washing, and scanning of all Affymetrix Genechips was performed according to standard Affymetrix protocols. Scans of the Affymetrix arrays were processed using the Affymetrix GeneTitan Instrument.

Quality control was performed on raw data by assessing the signal distribution by using scatter plot, MA-plot and a normal probability plot. Positive (landmark) and negative (blank) spots were used in the quality control and not used in further analyses. Normalized data were visualized by Principal Component Analysis (PCA) for additional quality assessment. The gene expression data have been deposited in NCBI's Gene Expression Omnibus (http://www.ncbi.nlm.nih.gov/geo/) and are accessible through GEO Series accession number GSE102016.

### 2.5 Analysis of microarray data

Microarray analysis was performed using the MADMAX pipeline for statistical analysis of microarray data (Lin et al., 2011). Briefly, microarrays were normalized with the robust multichip average method and probes were annotated according to Dai et al. (Bolstad et al., 2003, Dai et al., 2005). Individual genes were defined as changed when comparison of the normalized signal intensities showed a *p* ≤ 0.05 in a 2-tailed paired intensity-based moderated *t*-statistics (IBMT) and a fold change of > 1.2 or <− 1.2 (Sartor et al., 2006).

A first dedicated analysis focused on genes that were selected on basis of their known involvement in B[*a*]P metabolism (phase I and phase II enzymes), DNA repair and transport of B[*a*]P metabolites over the cell membrane (phase III). Meanwhile, comparison between the gene expression changes and phenotypic assays, which were published in previous work (Arlt et al., 2015), were made, such as Cyp1a (*i.e.* 7-ethoxy-resorufin-*O*-deethylase [EROD] and 3-cyano-7-ethoxycoumarin [CEC] assay), NAD(P)H:quinone oxidoreductase (Nqo1) and β-glucuronidase enzyme activity assays, NER capacity assay, and ^32^P-postlabelling for BPDE-DNA adducts. A second approach used PCA on all differentially expressed genes (DEGs) to identify the key variables to distinguish the four different treatments (*i.e.* control, LPS, B[*a*]P and LPS&B[*a*]P) (Raychaudhuri et al., 2000). These genes were subsequently analyzed for Gene Ontology (GO, http://www.geneontology.org/) and Kyoto Encyclopedia of Genes and Genomes (KEGG, http://www.genome.jp/kegg/pathway.html) pathway enrichment analysis by using the Database for Annotation, Visualization and Integrated Discovery (DAVID; version 6.8, http://david.abcc.ncifcrf.gov/summary.jsp) online software (Jiao et al., 2012). The GO function provides ontologies to attributes of gene function in three domains, including biological process (BP), molecular function (MF) and cellular component (CC) (Harris et al., 2004). The KEGG pathway analysis is a software which establishes pathway maps that contain current knowledge on biological networks (Zhang & Wiemann, 2009). Finally, an overall analysis was performed on the identified DEGs using DAVID.

### 2.6 Principal component analysis (PCA) analysis

Principal Components Analysis (PCA) was applied to separate samples of each treatment based on the gene expression and to visualize the distribution of the data. Before PCA analysis, a pool of 4731genes was pre-selected on basis of the statistical significance (*p* < 0.05) and fold change (>|1.2) between each type of treatment *versus* control. The data for these significant genes was visualized by PCA. Further reduction of the selected genes was done by calculating the PCA loadings, which assign a distance measure to each gene. A cut-off of 0.02 was used to select the genes with the highest norm value as they have the largest influence on the PCA distribution of the samples. Calculations were performed in the Matlab™ software package (The MathWorks, Inc., Natick, MA).

## 3 Results

### 3.1 Number of genes changed after each exposure

**Fig. 1:**
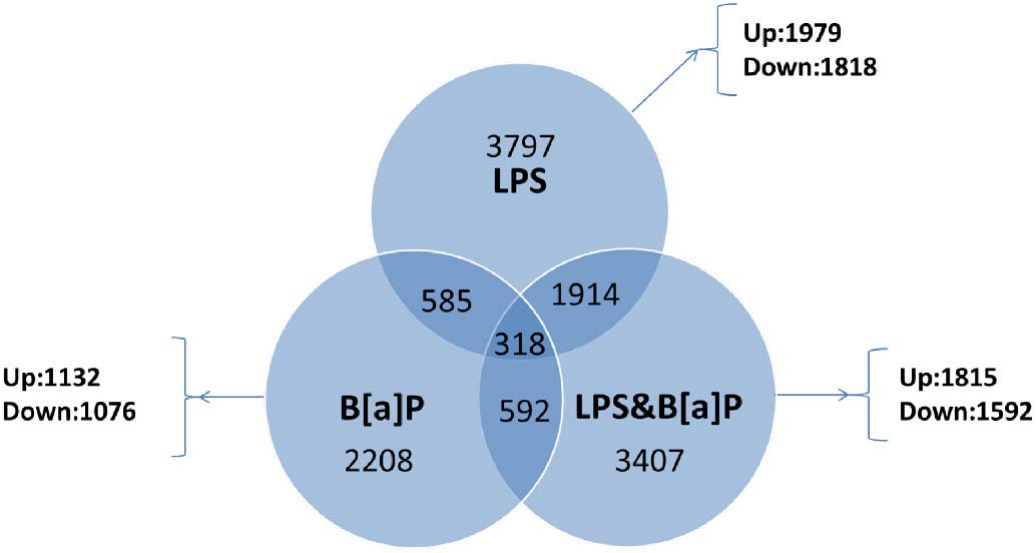
Venn diagram, where each circle shows the number of genes significantly expressed after different treatments (*e.g.* LPS, B[*a*]P and LPS with B[*a*]P) *versus* control.

When compared with control, a total of 3797, 2208 and 3407 genes were significantly (*p* ≤ 0.05, fold change > | 1.2 |) differentially expressed in LPS-exposed, B[*a*]P-exposed and LPS-combined with B[*a*]P-exposed mouse lungs, respectively (Fig. 1). Among the 2208 genes altered by B[*a*]P treatment, 1132 genes were up-regulated and 1076 genes were down-regulated. Of the 2208 genes that were differentially expressed after B[*a*]P exposure, 585 were also differentially expressed after LPS exposure, but not necessarily in the same direction. The combined exposure to B[*a*]P and LPS showed overlap with exposure to B[*a*]P only for 592 genes, but again not necessarily in the same direction. Finally, 318 genes were differentially expressed by all three treatments when compared to controls.

### 3.2 Dedicated analysis of gene expression profiles involved in B[*a*]P metabolism and DNA damage response

To gain further insight into the effect of LPS on B[*a*]P-induced carcinogenesis, a list of 57 genes were derived from published literature that indicated that these genes are involved in B[*a*]P metabolism, the cellular response to B[*a*]P and DNA repair (Kim et al., 1998, Ghosal et al., 2003, Moorthy et al., 2003, Arlt et al., 2015, Martignoni et al., 2006, Guo et al., 1994, Bauer et al., 1995, Luckert et al., 2013, Trush et al., 1991, Adams et al., 1995, Shi et al., 2009, Quinn and Penning, 2008, Kalabus et al., 2012, Stiborova et al., 2016b, Wada et al., 2013, Shen et al., 2010a, Iskander et al., 2005, Iskander et al., 2004, Zhang et al., 2011, Zheng et al., 2002a, Cai et al., 2010, Buckley and Klaassen, 2007, Shi et al., 2016a, Zhang et al., 2012, Shi et al., 2000, Romert et al., 1989, Raza et al., 1991, Alexandrov et al., 2002, Lodovici et al., 2004, Rojas et al., 2000, Saunders et al., 2006, Monari et al., 2007, Schults et al., 2013, Gungor et al., 2007, Kunze et al., 2015, Melis et al., 2013, Christmann and Kaina, 2013, Shen et al., 2008, Starostenko et al., 2016, Gibbons et al., 2014, Kranz et al., 2014. We divided the gene expression profiles into four categories, phase I metabolism, phase II metabolism, DNA damage response (DDR) and phase III reactions. In this analysis, we focused on the expression of LPS combined with B[*a*]P *versus* expression by B[*a*]P only to establish how LPS exposure can affect the B[*a*]P-induced gene expression changes. As shown in Table 1, 15 out of 17 genes belonging to phase I, phase II and DDR were significantly inhibited by additional exposure to LPS compared to B[*a*]P alone, including *Cyp1a1*, *Ephx1*, *Nqo2*, *Comt*, *Cat*, *Gss*, *Sult1a1*, *Gstp1*, *Gstm1*, *Gstt1*, *Gpx3*, *Sod3*, and *Ddb1*. Only *Xpa* and *Nox1* were up-regulated by additional exposure to LPS, when compared to B[*a*]P exposure only.

**Table 1:**
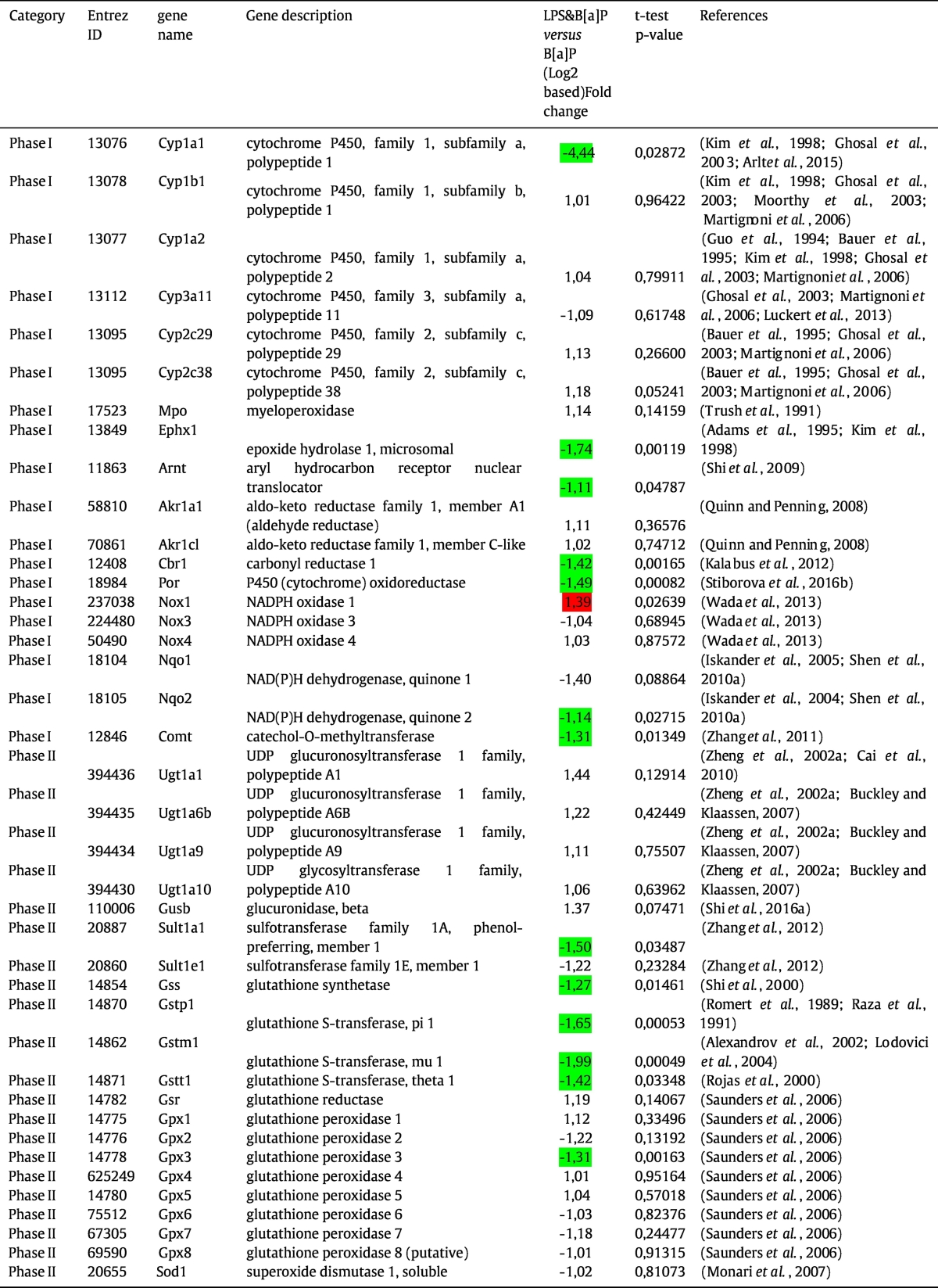

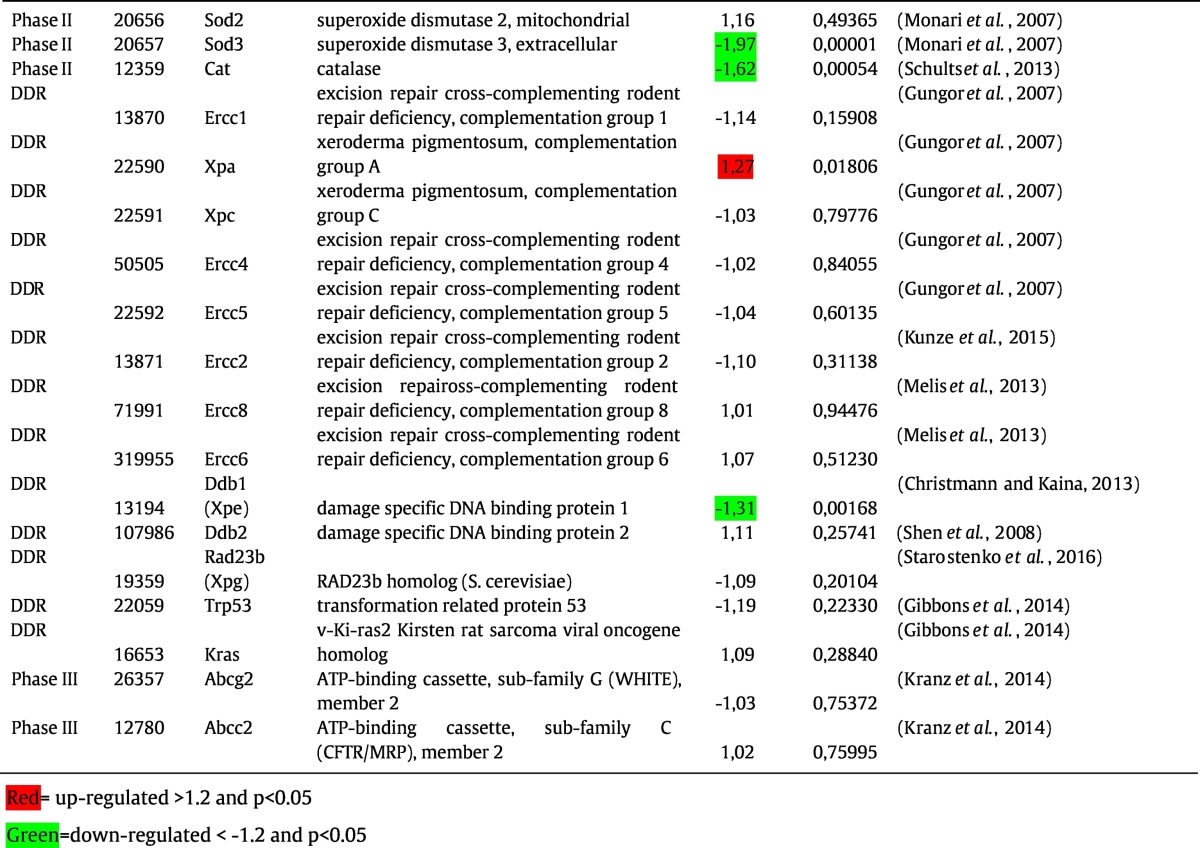
Summary of 57 genes that related to B[*a*]P metabolism and B[*a*]P-induced DNA damage.

### 3.3 Confirmation of DEGs by phenotypic assays

**Fig. 2:**
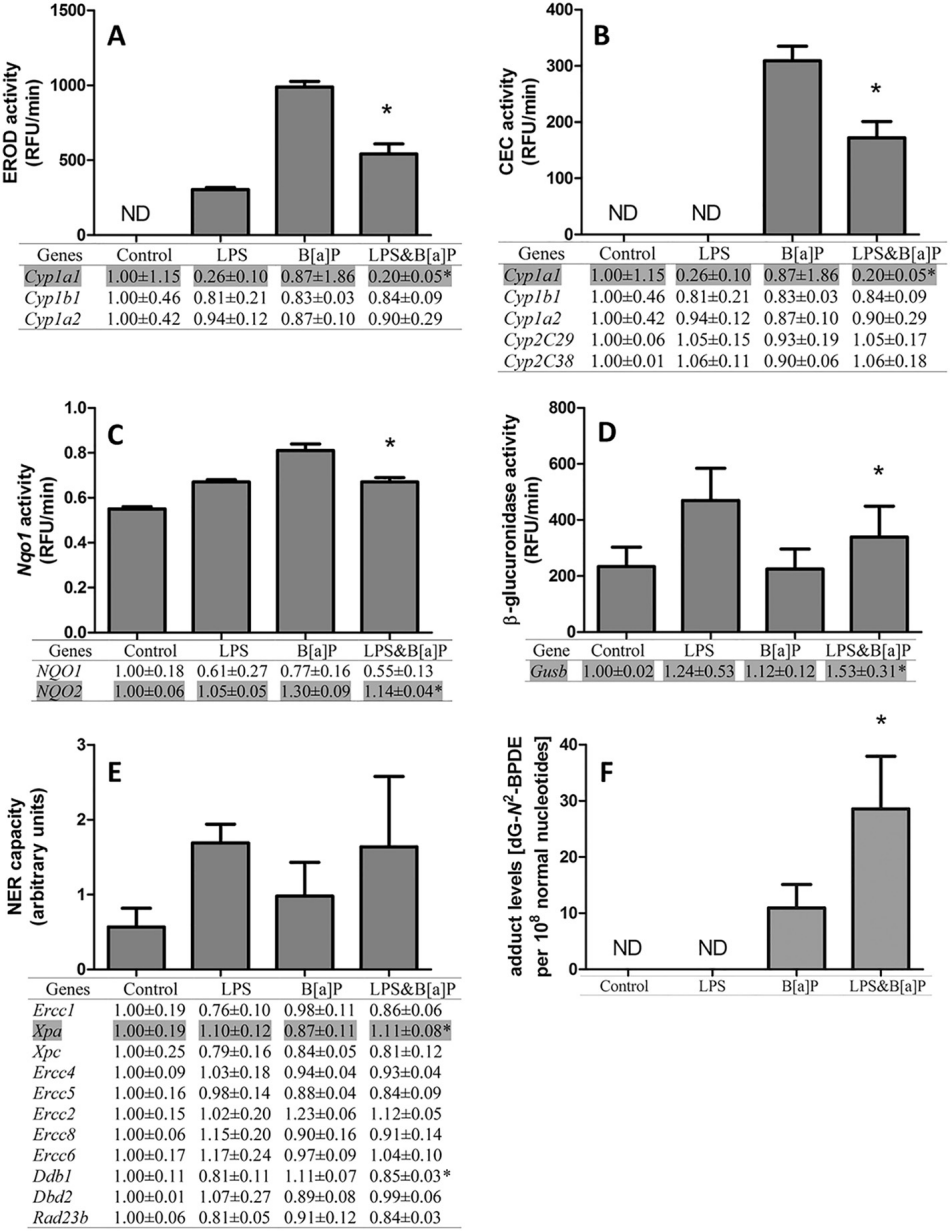
*In vitro* assay phenotypic data were partly derived from previously published work (Arlt et al., 2015, Shi et al., 2016a). Expression patterns of genes that are potentially relevant for each particular phenotypic assay are given underneath. (A) and (B) are EROD and CEC assay respectively, that mainly measure Cyp1a enzyme activity in microsomal fractions isolated from lung tissues of mice treated with vehicle (control), LPS, B[*a*]P and LPS&B[*a*]P, respectively. (C) Nqo1 enzyme activity in cytosolic fractions from lung tissues. (D) β-glucuronidase activity in cytosolic fractions from lung tissues. (E) NER repair capacity in tissue extracts isolated from lung tissues. (E) B[*a*]P-DNA adduct formation (*i.e.* dG-*N*^2^-BPDE) measured by ^32^P-postlabeling in lung of mice treated with vehicle (control), LPS, B[*a*]P and LPS&B[*a*]P. Expression data in different treatment groups was corrected by setting control values as 1. For each phenotypic assay, the best correlating gene was marked in grey color. All values are given as the means ± SD (*n* = 4). ND, not detected. RFU, relative fluorescence unit. Statistical analysis of the phenotypic assays was performed by 2-way ANOVA, followed by Tukey's multiple comparisons test, and the gene expression data were analyzed by a 2-tailed paired intensity-based moderated t-statistics (IBMT) (*p < 0 0.05 compared to mice treated with B[*a*]P only).

The expression of several key enzymes in B[*a*]P metabolism and the response to B[*a*]P appeared to be differentially expressed after additional exposure to LPS. In order to validate these results, the gene expression data were compared with several phenotypic assays, including the measurement of Cyp1a activity (EROD and CEC assay), Nqo1 activity, β-glucuronidase activity, and NER capacity (Fig. 2). Also B[*a*]P-DNA adduct levels were assessed as net result of B[*a*]P exposure, metabolism and DNA repair.

Gene expression of *Cyp1a1* correlated with Cyp1a enzymes activity as determined by both EROD and CEC assays (Fig. 2A and B).

Although gene expression of *Nqo1* was not significantly (*p* = 0.089) inhibited by LPS, Nqo1 enzyme activity was slightly lower (1.2-fold; *p* < 0.05) in LPS&B[*a*]P when compared to B[*a*]P exposure only. Interestingly, *Nqo2* mRNA expression was significantly down-regulated in the B[*a*]P&LPS group when compared to B[*a*]P exposure only, which may in part be reflected in this phenotypic assay (Fig. 2C).

β-Glucuronidase is produced and released by inflammatory cells after LPS exposure. Indeed, the β-glucuronidase activity was 1.5-fold higher in lungs of animals that were exposed to LPS&B[*a*]P, and similarly, the gene expression of *Gusb* was also 1.4-fold higher in LPS&B[*a*]P-exposed lungs compared to lungs exposed to B[*a*]P only (Fig. 2D).

Finally, NER capacity was assessed by a modified comet assay, and the expression of *Xpa* demonstrated the best correlation with this phenotypic endpoint (Fig. 2E). *Xpa* showed a 1.3-fold higher gene expression in LPS&B[*a*]P-exposed animals than after B[*a*]P exposure only. All other DNA repair enzymes did not have statistically significant changes in their expression; except for *Ddb1*, which showed a 1.3-fold lower expression in the LPS&B[*a*]P group compared to the B[*a*]P group. *Ddb1* was initially implicated in the process of NER, but later it was found that *Ddb1* primarily functions as a core component of E3 ubiquitin ligase complexes that regulate numerous essential processes in the cell, including DNA repair, DNA replication and chromatin remodeling (Fischer et al., 2014).

Of course mRNA expression does not always reflect phenotypic effects due to posttranslational modifications, but still most phenotypic assays in this study reflect the mRNA expression of their underlying genes. The relationship with DNA repair is more complicated, because of the involvement of many proteins in the NER process and NER is mostly post-translationally regulated. DNA repair activity was (non-significantly) up-regulated in the LPS&B[*a*]P group, which may be a direct response to increased levels of DNA damage. Indeed, B[*a*]P-DNA adduct levels after LPS&B[*a*]P exposure were 2.6-fold (*p* < 0.05) higher than after B[*a*]P exposure only (Fig. 2F).

### 3.4 PCA and KEGG pathway analysis

**Fig. 3:**
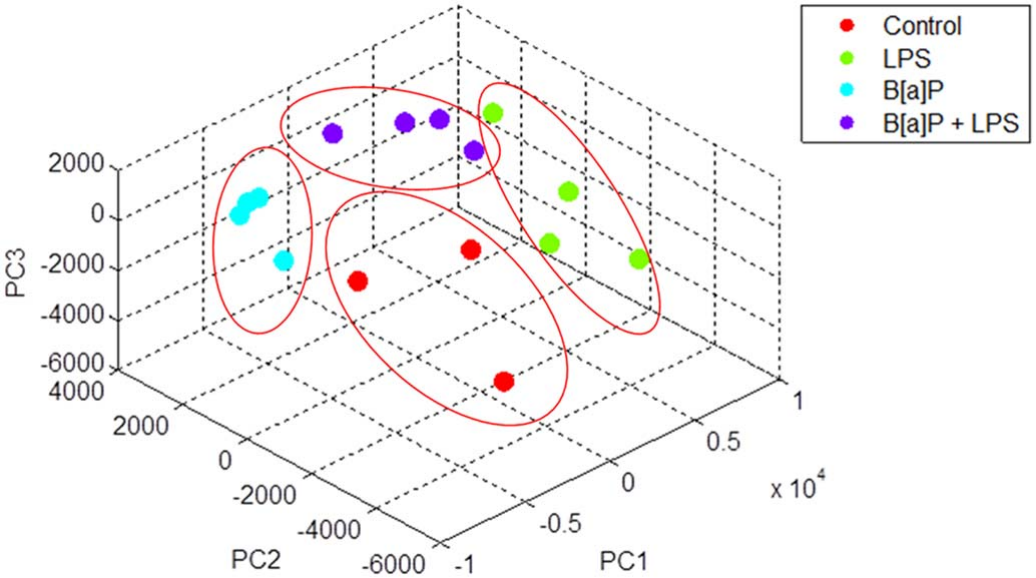
PCA analysis results of gene expressions that optimally differentiate between control, LPS, B[*a*]P and LPS&B[*a*]P treatment.

In order to investigate the interrelationship among all four groups of treatment, PCA was applied on all DEGs to identify those genes that could distinguish between the various treatments (Fig. 3). Of all the DEGs, 398 genes were identified that could differentiate between control, LPS, B[*a*]P and LPS&B[*a*]P group. To get further insights into the pathways in which these 398 genes are involved, KEGG pathway analysis was performed. As shown in Table 2, identified pathways related to the immune response and response to infections dominated the list of significantly altered pathways. Since the expression of these genes could also distinguish between control and B[*a*]P-exposed lung samples this indicates that B[*a*]P exposure alone can already affect inflammation and *vice versa*.

**Table 2:** KEGG pathway analysis of genes derived from PCA analysis.

Term	Count	%	Benjamini
Phagosome	23	5.8	4.40E − 08
Herpes simplex infection	22	5.5	3.80E − 06
Cell adhesion molecules (CAMs)	21	5.3	3.20E − 07
Tuberculosis	20	5	4.70E − 06
Regulation of actin cytoskeleton	20	5	6.30E − 05
Endocytosis	17	4.3	2.00E − 02
Leishmaniasis	16	4	1.10E − 08
Leukocyte transendothelial migration	16	4	1.50E − 05
Influenza A	16	4	6.00E − 04
Focal adhesion	15	3.8	9.10E − 03
*Staphylococcus aureus* infection	14	3.5	2.80E − 08
Measles	14	3.5	7.60E − 04
Antigen processing and presentation	13	3.3	3.10E − 05
Osteoclast differentiation	13	3.3	1.30E − 03
Viral myocarditis	11	2.8	6.20E − 04
Chagas disease (American trypanosomiasis)	11	2.8	3.50E − 03
Hepatitis C	11	2.8	2.20E − 02
Ribosome	11	2.8	3.20E − 02
Graft-*versus*-host disease	10	2.5	1.40E − 04
Type I diabetes mellitus	10	2.5	5.50E − 04
Adherens junction	10	2.5	1.30E − 03
Rheumatoid arthritis	10	2.5	2.90E − 03
Toxoplasmosis	10	2.5	2.20E − 02
Allograft rejection	9	2.3	1.30E − 03
Autoimmune thyroid disease	9	2.3	4.90E − 03
Pertussis	9	2.3	6.20E − 03
Toll-like receptor signaling pathway	9	2.3	3.20E − 02
Arrhythmogenic right ventricular cardiomyopathy (ARVC)	8	2	2.10E − 02
Complement and coagulation cascades	8	2	2.70E − 02
Prion diseases	7	1.8	1.80E − 03
Inflammatory bowel disease (IBD)	7	1.8	3.00E − 02
Biosynthesis of unsaturated fatty acids	5	1.3	3.20E − 02

### 3.5 Overall analysis of impact of LPS and B[*a*]P exposure: identification of DEGs

**Fig. 4:**
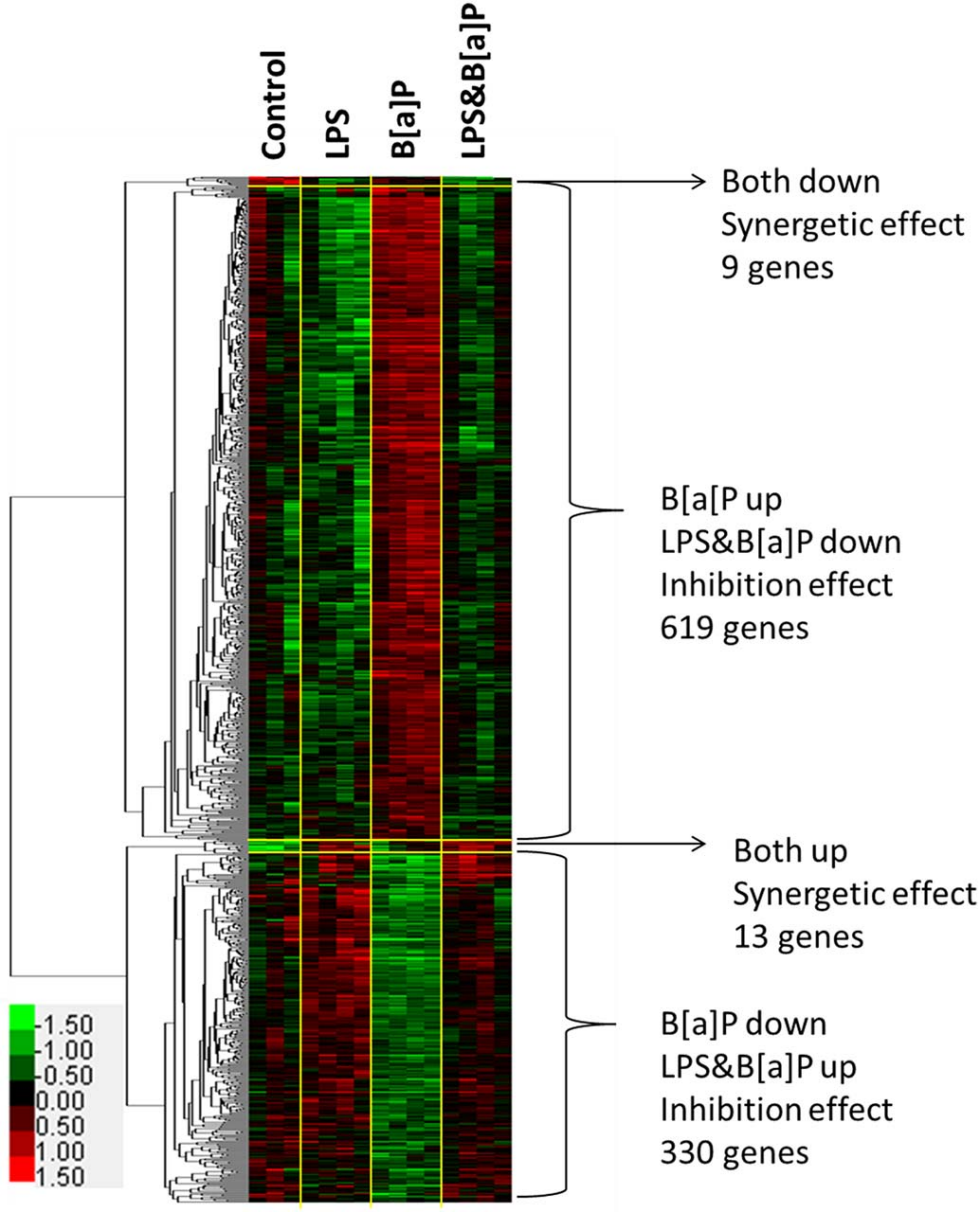
Hierarchical clustering of genes in Control, LPS, B[*a*]P and LPS & B[*a*]P treated samples. In hierarchical clustering, the red color represents up-regulation and the green denotes down-regulation. (For interpretation of the references to color in this figure legend, the reader is referred to the web version of this article.)

Finally, to investigate the effect of LPS on B[*a*]P-induced genotoxicity, an unsupervised analysis was performed. DEGs had to have a *p* ≤ 0.05 in a 2-tailed paired intensity-based moderated *t*-statistics (IBMT) and a cut-off of fold change of >|1.2 |. We filtered data and identified DEGs by using the following criteria: 1) Genes are significantly differentially expressed in B[*a*]P-exposed *versus* control animals; and 2) Genes are additionally significantly differentially expressed in the LPS&B[*a*]P group *versus* B[*a*]P group. In other words, we focused on genes that are differentially regulated by B[*a*]P and their expression is subsequently altered by the presence of inflammation. With these selection criteria, the DEGs that were identified will not represent an LPS effect. This resulted in a total of 971 DEGs (Fig. 4). 9 genes were down-regulated after B[*a*]P exposure when compared to controls and further significantly down-regulated by the combined exposure to LPS and B[*a*]P. Similarly, 13 genes were up-regulated by the B[*a*]P group and further significantly up-regulated in the LPs&B[*a*]P group. In total, these 22 genes showed a synergistic effect of the combined treatment. However, most DEGs (949 genes) demonstrated an inhibition in the combined exposure. For example, 619 genes were significantly up-regulated after B[*a*]P exposure when compared to controls, but these genes were again significantly down-regulated in the LPS&B[*a*]P group. Similarly, 330 genes were significantly down-regulated in the B[*a*]P group compared to control, whereas up-regulated in combination with LPS treatment.

**Fig. 5:**
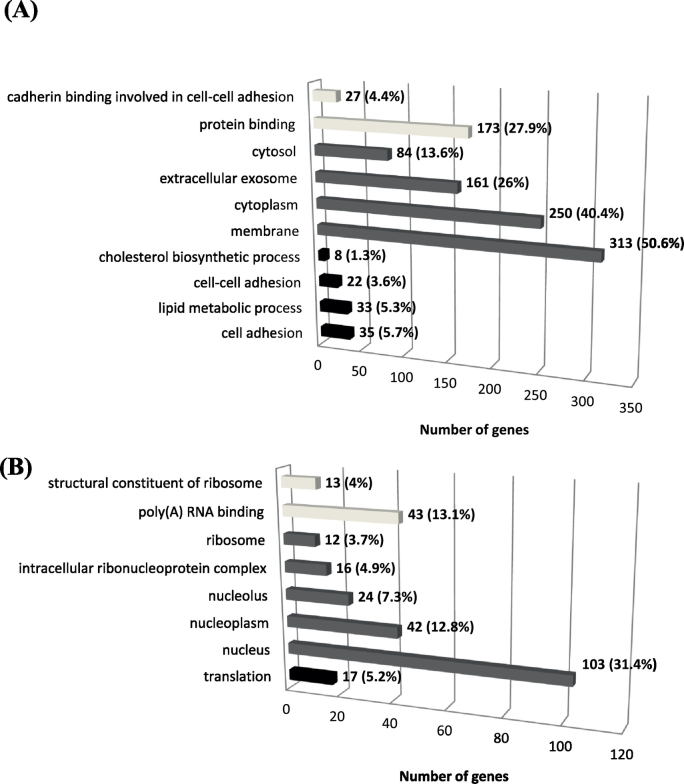
The numbers of genes and the percentage over whole gene set in each enriched GO terms that were identified for group 1 gene set (619 genes) (A) and group 2 gene set (330 genes) (B). Dark bar represents Biological Process (BP); Grey bar represent (Cellular Component) and light grey represent Molecular Function (MF).

**Table 3:** KEGG pathway enrichment analysis of DEGs.

Term	Count	%	Benjamini
Functional annotation for 619 genes			
Pathways in cancer	26	4.2	2.40E − 02
Endocytosis	24	3.9	2.40E − 03
Proteoglycans in cancer	17	2.7	2.60E − 02
Focal adhesion	16	2.6	4.70E − 02
Axon guidance	14	2.3	1.60E − 02
Adherens junction	12	1.9	3.20E − 03
ECM-receptor interaction	11	1.8	2.00E − 02
N-Glycan biosynthesis	8	1.3	2.30E − 02
Acute myeloid leukemia	8	1.3	4.50E − 02
Steroid biosynthesis	6	1	1.40E − 02
Thyroid cancer	6	1	4.40E − 02
Functional annotation for 330 genes			
Ribosome	10	3	8.80E − 03

### 3.6 Functional and pathway enrichment analysis

Due to the limited number of genes that were identified in the ‘synergistic effect’ gene set (22 genes), further analysis focused on the ‘inhibitory effect’ gene set (949 genes). We assigned the 619 genes that were up-regulated by B[*a*]P but their increased expression was subsequently inhibited by LPS as group 1, and the other 330 genes as group 2. The list of 949 genes that were identified were analyzed using DAVID to further explore the functions of these DEGs. The top 4 GO terms in each Biological Process (BP), Cellular Component (CC) and Molecular Function (MF) are presented in Fig. 5. The group 1 gene set (619 genes) was significantly enriched in cell adhesion (35 [number of genes involved], 6% [the percentage over whole gene set]), protein binding (173, 28%), cytoplasm (250, 40%), extracellular exosome (161, 26%), membrane (313, 51%), and lipid metabolic process (33, 5%) (for complete analysis see Supplementary Data 1). The results for group 2 (330 genes) were as follows: translation (17, 5.2%), poly(A) RNA binding (43, 13.1%) and nucleus (103, 31.4%) (for complete analysis see Supplementary Data 2). The same groups were used for pathway analysis by KEGG (Table 3). Group 1 genes were mainly involved in pathways associated with adherence junction, endocytosis, focal adhesion, *N*-glycan biosynthesis, acute myeloid leukemia, steroid biosynthesis, extracellular matrix (ECM)-receptor interaction, and axon guidance. In addition, pathways related to cancer were also observed for the group 1 gene set. On the other hand, only ‘ribosome’ (*p* = 0.0088) was identified in the KEGG pathway analysis for the group 2 gene set. Thus, both analyses (GO terms and KEGG) suggest that cell-cell adhesion and the extracellular matrix is modulated by the combined exposure to B[*a*]P and LPS when compared to B[*a*]P exposure only.

## 4 Discussion

It has been extensively described that B[*a*]P forms DNA adducts in mouse lung which are involved in lung carcinogenesis (Zuo et al., 2014). More recently, it has become clear that inflammation can further increase the genotoxicity of B[*a*]P (Arlt et al., 2015). Because inflammatory signaling is complex, and the fact that inflammation can impact on carcinogenesis at multiple levels, there is a lack of complete understanding of how inflammation affects B[*a*]P-induced carcinogenesis and therefore more knowledge is needed to effectively intervene in the process. To get more insight into the processes that are altered by B[*a*]P exposure during acute pulmonary inflammation, we used microarray technology in a mouse model to identify gene expression patterns that were affected by both stimuli. The resulting gene expression profiles were additionally compared with results of our previous studies (Arlt et al., 2015, Shi et al., 2016a, Shi et al., 2016b, Shi et al., 2017) which have measured multiple molecular endpoints to further increase our biological understanding of the influence of LPS on B[*a*]P-induced genotoxicity.

It is known that B[*a*]P by itself is not genotoxic because it does not contain active groups in the molecule, but it's reactive metabolites may contain highly reactive groups that can bind covalently to macromolecules (*e.g.* protein and DNA). A variety of enzymes are involved in B[*a*]P metabolism, including enzymes encoded by *Cyp1a1*, *Cyp1b1*, *Ephx* or *Arnt* (Hamouchene et al., 2011, Hockley et al., 2007, Zuo et al., 2014). Since a lot of information is already available about the metabolic pathways of B[*a*]P, targeted analysis of gene expression profiles was performed, in which we focused on genes that are known to be involved in B[*a*]P metabolism and B[*a*]P-induced DNA damage response. A total of 57 genes were selected based on studies previously published (Table 1). We directly compared the expression of these genes in the B[*a*]P and LPS&B[*a*]P treatment group and showed that gene expression of several key enzymes in phase I metabolism (*e.g. Cyp1a1*, *Ephx1*, *Arnt*, *Cbr1*, *Por*, *Nqo2* and *Comt*) were significantly inhibited by additional exposure to LPS. This may theoretically lead to slower metabolism of B[*a*]P. On the other hand, exposure to B[*a*]P is reported to cause gene up-regulation of various phase II detoxification enzymes, including GSTs, UGTs and SULTs (Gelboin, 1980). In our study gene expression of a majority of these phase II detoxification enzymes was significantly down-regulated by prior exposure to LPS, including *Sult1a1*, *Gstp1*, *Gstm1*, *Gstt1* and *Gpx3*. In contrast our results indicated that UGTs like *Ugt1a1* and *Ugt1a6* were all up-regulated by prior exposure to LPS although these changes were not statistically significant; UGTs are major detoxification enzymes catalyzing the conjugation of B[*a*]P-7,8-dihydrodiol to glucuronides. B[*a*]P-glucuronides can be cleaved by β-glucuronidase (*Gusb*). Interestingly, although *Gusb* gene expression was not statistic significantly up-regulated (*p* = 0.075) β-glucuronidase enzyme activity was significantly up-regulated in animals that were treated with both B[*a*]P and LPS. Therefore, β-glucuronidase could convert glucuronided B[*a*]P metabolites back into their active forms (Shi et al., 2016a). Due to the down-regulation of the phase II enzymes, cells can subsequently not sufficiently detoxify the active metabolites, potentially leading to more DNA adducts. Indeed, higher levels of B[*a*]P-induced adducts were found in inflamed lungs after B[*a*]P exposure.

In addition, *Nox1* was significantly up-regulated in the LPS&B[*a*]P group. *Nox1* is a biomarker for cellular oxidative stress, indicating that more ROS are produced in LPS&B[*a*]P-exposed animals, which may cause additional DNA damage (Knaapen et al., 2006). It was previously reported that ROS may inhibit NER (Hakem, 2008). However, in the present study, out of the 11 selected NER-related genes, only *Xpa* and *Xpe* were significantly up-regulated and down-regulated by LPS, respectively. In this case, gene expression changes did not necessarily reflect repair activity, probably because DNA repair activity is mainly regulated at the post-translational level.

To visualize the potential phenotypic effects of gene expression changes, we linked the gene expression profiles with phenotype data which were published by us in our previous studies (Fig. 2). The EROD and the CEC assay both mainly measure the activity of CYP1 family enzymes (Krais et al., 2016, Martin et al., 2010). Using the EROD and CEC assay we observed a significant inhibitory effect in the LPS&B[*a*]P-treated animals compared to animals treated with B[*a*]P only; *Cyp1a1* expression changes observed here correlated with the phenotype. In contrast to Cyp1a1 gene expression and enzyme activity, the quantitation of Cyp1a1 and Cyp1b1 proteins by Western blotting showed no difference between B[*a*]P- and LPS&B[*a*]P-exposed mouse lungs (previously published data; (Arlt et al., 2015)). At this moment it is unknown why gene expression and protein levels of these Cyp1 enyzmes do not represent Cyp1 activity. One possible reason is the selection of a single time point (48 h) for both measurements; Cyp1a1 gene expression is an early event whereas protein expression would be considered a later event and enzyme activity may additionally be modulated by posttranslational protein modification. Moreover, Cyp activity can also be inhibited by ROS (Karuzina and Archakov, 1994a, Karuzina and Archakov, 1994b, Morel and Barouki, 1998), which is produced in excess by exposure to LPS, which may provide an explanation why protein levels and enzyme activity do not correlate.

In addition, *Cyp1a1* is the most induced CYP enzyme after B[*a*]P exposure in both *in vitro* and *in vivo* studies, and it is most relevant for B[*a*]P metabolism (Arlt et al., 2008, van Delft et al., 2010, Stiborova et al., 2016a). However, the role of Cyp enyzmes in B[*a*]P-induced genotoxicity is still not fully elucidated. While in *in vitro* studies increased activity of Cyp1a1 is predominantly involved in the activation of B[*a*]P, paradoxically in *in vivo* studies it seems that Cyp1a1 is more important for B[*a*]P detoxification (Arlt et al., 2008, Arlt et al., 2012). Since the current study was an *in vivo* experiment, a decreased expression of *Cyp1a1*/Cyp1a1 by the presence of inflammation might indicate that less B[*a*]P is detoxified. Also some for other phenotypic assays, the gene expression pattern did not correspond directly to activity (*i.e.* NER and NQO1 activity). NER is the major repair mechanism for B[*a*]P induced DNA damage and the measured repair activity is the net effect of a combination of enzymes during the NER process (recognition and incision) (including *Xpa*, *Xpc*, *Ercc1*, *Ercc2*, and *Ercc 4*) (Schärer, 2013). NER capacity was higher in the LPS&B[*a*]P group than in the B[*a*]P-exposed animals or controls, but it did not reach statistical significance. Enhanced NER activity seems logical as a reaction to B[*a*]P-induced DNA damage, but due to the LPS-induced oxidative stress both expression of some NER-related genes (Fig. 2E) and activity can be inhibited (Langie et al., 2007). Since the level of DNA damage is determined by the formation of DNA adducts and their removal, the higher levels of DNA damage that were observed in the LPS&B[*a*]P group cannot be related to changes in DNA repair activity alone.

NQO1 can protect cells against oxidative stress that is induced by redox cycling of B[*a*]P-quinones (Shen et al., 2010b). The B[*a*]P-induced activity of *Nqo1* was significantly inhibited by LPS. However, in the current study we found that *Nqo2* gene expression displayed a better correlation than *Nqo1* gene expression with the phenotypic measure of Nqo1 enzyme activity. It is possible that the phenotypic assay is not specific for Nqo1 alone, but may additionally reflect Nqo2 enzyme activity. Interestingly, higher gene expression of *Nox1* and lower gene expressions of *Nqo1* and *Nqo2* might lead to higher levels of oxidative stress in LPS&B[*a*]P animals than animals treated with B[*a*]P or even LPS alone, further driving the inflammatory response. Indeed, PCA analysis indicated that 398 genes could differentiate animals among all treatment groups (control, LPS, B[*a*]P and LPS&B[*a*]P), and most of these genes are known to be involved in immune response and inflammation (Table 2). Interestingly, both B[*a*]P and BPDE have been reported to induce inflammation and immune responses in both *in vitro* and *in vivo* studies (Dreij et al., 2010, Qamar et al., 2012). These data suggest that genes involved in inflammation may be key determinants in B[*a*]P-induced genotoxicity.

To further analyse the data in an unsupervised approach we identified DEGs on the basis of 2 criteria: 1. They were altered in expression by B[*a*]P exposure and 2. The additional exposure to LPS modified their gene expression. This resulted in genes that mainly displayed an inhibitory effect (*i.e.* gene expression initially changed by B[*a*]P but again altered towards controls in the presence of inflammation), and we subsequently performed GO and KEGG enrichment analysis on gene set group 1 (619 genes, initially up-regulated by B[*a*]P) and 2 (330 genes, initially down-regulated by B[*a*]P) *via* the online tool DAVID. Among the GO terms identified for the Group 1, the majority of genes were involved in cellular components: membrane (313 genes), cytoplasm (250 genes) and extracellular exosome (161 genes). These cellular components are essential for maintaining cellular intactness and interaction between cells (Edgar, 2016, Tekpli et al., 2010). Many of these genes were related to cell adhesion. Cell adhesion plays a significant role in inhibiting the processes in multistage carcinogenesis, cancer cell local invasion and metastasis (Bremnes et al., 2002). However, our data indicate that inflammation (induced by LPS treatment) inactivated cell adhesion, which may further stimulate lung cancer development that was initially induced by B[*a*]P. For the group 2 gene set (initially down-regulated by B[*a*]P), genes related to ‘RNA translation’ processes were identified. This was unexpected because it is known that B[*a*]P can enhance its own bio-activation by inducing gene expression (*e.g. Cyp1a1*, *Cyp1b1* and *Mpo*) *via* binding to xenobiotic-response elements in the nuclear DNA (Schults et al., 2013). This should lead to increased RNA production and subsequent translation. It is possible that part of the phenotypic inhibition is due to a slower translation of mRNA into functional proteins. However, it should be noted that we measured RNA expression at 48 h after B[*a*]P exposure, and at that time point the largest part of B[*a*]P may already be metabolized and removed. LPS inhibited the metabolism of B[*a*]P and therefore we might detect a delayed effect of B[*a*]P in the lung in the presence of LPS. Another possibility could be that LPS inhibited the down-regulation of genes involved in RNA translation.

The KEGG pathway enrichment analysis showed similar results as the analysis of GO terms. For example, pathways involved in endocytosis and adherens junction were identified for the group 1 gene set and pathways involved in ribosome function was found for the group 2 gene set. Besides that, several pathways related to cancer were observed for group 1 genes. Specifically, genes related to cytokine-cytokine receptor interaction, Wnt signaling pathway and mitogen-activated protein kinase (MAPK) signaling pathway were found. Moreover, the endpoint of these pathways all point to cell proliferation and differentiation. This might provide additional evidence for the delayed effect of B[*a*]P due to prior LPS exposure; after exposure to B[*a*]P for 48 h, cells are recovering from the B[*a*]P-induced damage by modulating cell cycle, DNA repair, and cell proliferation and differentiation (van Delft et al., 2010). But, in the presence of LPS, all of these normal cellular responses after B[*a*]P exposure are inhibited and may happen at or later than 48 h after the exposure to B[*a*]P.

The current study has some limitations that need to be mentioned. Firstly, the RNA microarray was performed on RNA derived from whole mouse lung. Therefore, part of the gene expression changes may be related to the influx of inflammatory cells into the lung. For instance, increased expression and activity of beta-glucuronidase is probably not due to increased expression of this gene in lung epithelial cells, but may be due to the fact that activated inflammatory cells expressing this gene are present in higher amounts in the lung after LPS exposure. Nevertheless, the gene expression changes observed in the current study still give an indication for the general underlying mechanism(s) and help to understand why the presence of inflammation in the lung leads to higher levels of DNA damage. Secondly, the effect of LPS treatment was not directly included in in the selection of DEGs. Therefore, some of the DEGs that were selected based on our criteria may be related to an LPS effect only. Indeed, we showed that over 50% of the DEGs in the LPS and LPS&B[*a*]P group overlap (Fig. 1). However, since it was not the focus of our study to investigate a pure LPS effect on gene expression, these data are not shown but all gene expression data have been deposited online and can be accessed.

Last but not least, the animals were sacrificed at one single time point after exposure. Indeed, 48 h may be too late for assessing very early B[*a*]P metabolic events, but in our previous studies in which we exposed rodents to B[*a*]P, highest DNA adduct levels were always found at approximately 2 days after the exposure (Arlt et al., 2015). Therefore, to investigate cellular effects at the highest level of DNA damage, 48 h was chosen as exposure time point.

## 5 Conclusion

**Fig. 6:**
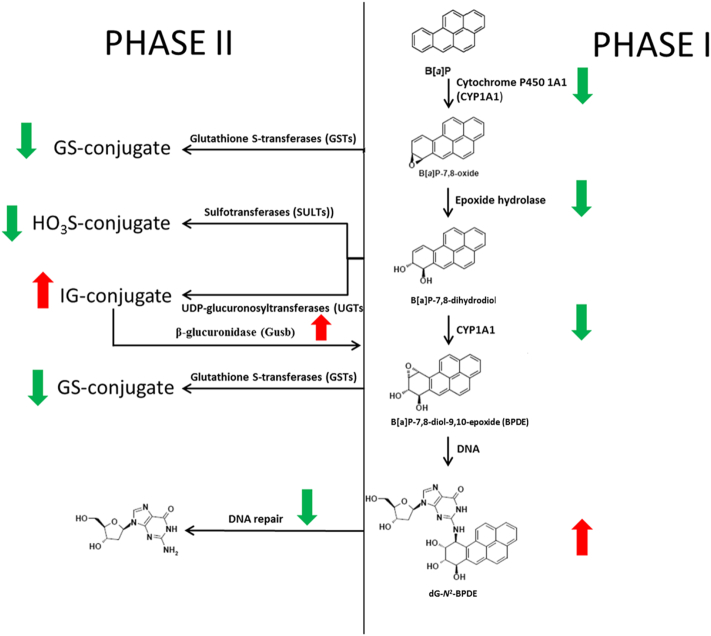
Proposed alternation of B[*a*]P metabolism pathway after initial LPS-induced inflammation. This pathway is based on our transcriptome analysis together with previous phenotypic analyses, suggesting that LPS inhibits *Cyp1a1*, epoxide hydrolase, GSTs, SULTs. Although UGTs are up-regulated by LPS, β-glucuronidase, which could convert UGTs conjugated Ba]P metabolites into their free form, is also up-regulated. Finally, LPS enhanced the B[*a*]P-induced DNA damage by forming BPDE-DNA adducts.

Many previous microarray studies have investigated the effect of B[*a*]P exposure *in vitro* and *in vivo*, and we confirmed these results by showing that B[*a*]P can disrupt cholesterol (steroid) metabolism, DNA repair and cell cycle at 48 h after exposure. The additional treatment to LPS is less well studied, but may mimic real life situations much better, because many types of environmental exposures (*e.g.* cigarette smoke, vehicle exhaust, ambient air particulate matter) not only result in the exposure to PAHs but are also capable of inducing inflammation. Combined exposure of mice to LPS and B[*a*]P resulted in a complex gene expression response which pointed towards delayed metabolism of B[*a*]P. Although the expression of genes that code for enzymes that are known to activate B[*a*]P were inhibited by inflammation, genes of enzymes in phase II detoxification reactions were also down-regulated by LPS (Fig. 6). Consequently, exposure to LPS seems to slow down B[*a*]P metabolism and this leads to a prolonged exposure of lung cells to B[*a*]P. As a consequence higher B[*a*]P-DNA adduct levels were observed in mouse lung after 48 h of exposure. Additionally, our analysis indicated that cell-cell communication is disturbed which is important for the process of lung carcinogenesis. Overall, our data support the idea that the combined exposure to PAHs and inflammation will lead to an increased risk for developing lung cancer.

## Conflict of interests

The authors declare that there is no conflict of interest.

## Appendix A Supplementary data

Supplementary Data 1 The DAVID GO terms analysis of gene set group 1 (619 genes). Supplementary Data 1

Supplementary Data 2 The DAVID GO terms analysis of gene set group 2 (330 genes). Supplementary Data 2
